# Draft Genome Sequence of *Bacillus thuringiensis* var. *thuringiensis* Strain T01-328, a Brazilian Isolate That Produces a Soluble Pesticide Protein, Cry1Ia

**DOI:** 10.1128/genomeA.00817-13

**Published:** 2013-10-10

**Authors:** Alessandro M. Varani, Manoel V. F. Lemos, Camila C. Fernandes, Eliana G. M. Lemos, Eliane C. C. Alves, Janete A. Desidério

**Affiliations:** Departamento de Tecnologia, Universidade Estadual Paulista “Júlio de Mesquita Filho,” Campus de Jaboticabal, São Paulo, Brazila; Departamento de Biologia Aplicada à Agropecuária, Universidade Estadual Paulista “Júlio de Mesquita Filho,” Campus de Jaboticabal, São Paulo, Brazilb

## Abstract

*Bacillus thuringiensis* var. *thuringiensis* strain T01-328, isolated from Cubatão county (São Paulo State, Brazil), produces a soluble pesticide protein, Cry1Ia, during vegetative growth. Here, we report the 7.089-Mbp draft genome sequence, composed of a 5.5-Mb chromosome and 14 plasmids, which is the largest *B. thuringiensis* genome sequenced to date.

## GENOME ANNOUNCEMENT

*Bacillus thuringiensis* is an important and widespread biological control agent that has the ability to sporulate and produce parasporal bodies (1). These parasporal bodies harbor the crystal (Cry) protein, which acts as a defense system against pests that cause enormous losses in crop production (2). At the same time, this bacterium has an array of other genetic information which codes for vegetative insecticidal proteins, which control pests in a different fashion (3). Together, these proteins, when inserted into the genomes of important crops, increase plant defenses to prevent the loss of productivity (4, 5). *B. thuringiensis* exhibits a wide variety of extrachromosomal elements (6) and the genetic information for these insecticidal proteins may be distributed among these elements and in the chromosome itself. The *B. thuringiensis* Cry proteins are commonly nonsoluble and produced during sporulation. In contrast, the T01-328 strain produces a soluble Cry1Ia crystal during vegetative growth.

*B. thuringiensis* var. *thuringiensis* strain T01-328 was collected in 1994 from soil samples in Cubatão county, located in the eastern part of São Paulo, Brazil (23°53′43″S, 46°25′32″W). During the 1980s, Cubatão was considered one of the most polluted cities in the world due the activities of oil refining and fertilizer industries. Although measures were taken by the Brazilian government to diminish the city’s pollution, it is uncertain whether the soil and underground water can be completely cleaned.

The genome sequencing of strain T01-328 was carried out using the SOLiD 5500xl and Illumina HiScan platforms. A total of 180 million mate-pair reads with an insert size of 3 kb (SOLiD) and 60 million paired-end reads with an insert size of 220 bp (Illumina) were generated and provided ~1,500-fold coverage of the genome. *De novo* assembly was carried out using the CLC Genomics workbench 6.0.5 (CLC bio, Aarhus, Denmark). The assembled sequences were linked into 143 scaffolds using SSPACE (7) and GapFiller (8). By use of SIS (10) and CONTIGuator (11), the obtained scaffolds were mapped against the complete sequences of other *B. thuringiensis* strains deposited in public databases. The genome sequence has a total size of 7,089,686 bp and a G+C content of 34.37%, which represents the largest sequenced genome of *B. thuringiensis* to date, according to the Genomes OnLine database (GOLD) (9). The T01-328 genome consists of a 5.5-Mb circular chromosome and 14 plasmids with estimated sizes of 2.2 kb, 5.7 kb, 7 kb, 7.6 kb, 8.4 kb, 8.9 kb, 12 kb, 68 kb, 80 kb, 96 kb, 215 kb, 285 kb, 292 kb, and 501 kb. Interestingly the 501-kb megaplasmid is 99.99% identical to the BTB_502p from *B. thuringiensis* 407 Cry-, previously reported as novel and as the largest *B. thuringiensis* plasmid found to date (12). The annotation was performed using the Prokka pipeline (Prokaryotic Genome Annotation System), which predicted 7,403 coding sequences. Genome analysis revealed that the T01-328 *cry1Ia* gene is located on the 285-kb megaplasmid and is 100% identical to the nonsoluble *cry1Ia* gene from *B. thuringiensis* subsp. *kurstaki* (Uniprot Q45752). A comparative genomic analysis concerning the impact that the modified environmental conditions of Cubatão county have had on *B. thuringiensis* strain T01-328 will follow in a future publication.

### Nucleotide sequence accession numbers.

The annotated sequence of the *B. thuringiensis* var. *thuringiensis* T01-328 has been deposited at DDBJ/EMBL/GenBank under the accession number ARXZ00000000. The version described in this paper is version ARXZ02000000.
